# Personalized tricuspid valve modeling predicts regurgitation biomechanical repair and double‑orifice flow

**DOI:** 10.1007/s13246-026-01740-4

**Published:** 2026-05-11

**Authors:** Giuseppe Sausa, Giovanni Gentile, Nicola Cuscino, Salvatore Pasta

**Affiliations:** 1 Department of Research, IRCCS ISMETT via Tricomi, Palermo, Italy; 2 Radiology Unit, Department of Diagnostic and Therapeutic Services, IRCCS ISMETT, via Tricomi, 5, Palermo, Italy; 3 Department of Engineering, Università degli Studi di Palermo, Viale delle Scienze Ed.8, Palermo, Italy

**Keywords:** Tricuspid valve, Tricuspid regurgitation, MitraClip device, Finite-elements method, Lattice-boltzmann method

## Abstract

Tricuspid regurgitation (TR) is a prevalent and progressive condition associated with heart failure symptoms; however, numerous patients are unsuitable for surgery, and achieving a long-term outcome is challenging. This renders TR an unmet clinical problem. Computational modeling facilitates the evaluation of the biomechanics associated with regurgitation, aiming to enhance therapeutic strategies as transcatheter edge-to-edge repair. This study sought to establish a computational framework integrating a tricuspid valve (TV) model with the right ventricle (RV) wall to evaluate native valve function, TR pathology, and repair using the MitraClip device. Computed tomography angiography (CTA) from three patients was utilized to reconstruct the tricuspid valve leaflets, annulus, and RV wall. A parametric CAD pipeline for modeling the leaflet surfaces and chordal architecture was built using ex-vivo data. Annular kinematics (systole-to-diastole) were computed and then applied as a boundary condition in the finite-element analyses, with tricuspid regurgitation induced by displacing papillary positions and augmenting transvalvular loading. The MitraClip deployment was subsequently simulated whereas the post-deformation hemodynamics were assessed using a lattice-Boltzmann solver to quantify intraventricular velocity fields and vorticity. All three models demonstrated physiological leaflet coaptation and stress distributions in a healthy patient condition, with maximum stresses at end-systole quantified in the leaflet belly and chordal insertions. Post-MitraClip simulations demonstrated restored valve closure without retrograde flow during systole; however, flow exhibited a double-orifice inflow and localized vorticity near the clip during diastole. The jet orientation and downstream patterns depended on patient anatomy. This computational framework replicates TV biomechanics and post-repair hemodynamics from standard imaging, facilitating quantitative, patient-specific evaluation. The current patient-specific, image-based computational method can predict TV-related performance and post-repair hemodynamic outcomes, facilitating personalized planning to minimize residual TR and enhance device placement.

## Introduction

The tricuspid valve (TV), which regulates blood flow between the right atrium and right ventricle (RV), is composed of an annulus, characteristically saddle-shaped in healthy conditions, and three main leaflets: the anterior (the largest), septal and posterior. Tricuspid valve regurgitation (TR) affects more than 1.6 million Americans [1], but classification of morphological and functional TR is essential for assessing the optimal therapeutic strategy. Primary TR due to sub-valvular dysfunction is observed in only 8–10% of the entire patient population [2]; however, the majority of cases arise in individuals with left-sided heart diseases. Indeed, secondary TR is likely caused by functional consequence of the left heart chambers and a pressure increase in the pulmonary circulation [3]. Volume overload and increased wall stress in the right-sided heart chambers negatively contributes to adverse remodeling and worsening. As TR becomes more severe, right ventricular dilatation occurs by leading to right ventricular failure and ultimately adverse outcomes. However, medical management remains poor with a 5-year mortality approaching 50% [1]. Surgical TV repair is the preferential treatment option, but the optimal timing for elective surgery remains unclear. Recently, transcatheter tricuspid valve interventions with edge-to-edge repair [4], valve devices [5, 6] or leaflet approximations [7] have been developed for selected patients. Though the MitraClip device (Abbott Vascular, Santa Clara, USA) is designed for the mitral valve, the confidence matured by interventional cardiologists has been considered as advantageous compared to other structural approaches when treating either primary or secondary TR [8]. The procedure can be carried out under echocardiographic guidance with not any relevant changes in the device design.

A comprehensive understanding of TV biomechanics is essential for enhancing current repair methods and formulating novel therapeutic approaches. A further challenge relies in the considerable inter-patient variability of tricuspid valve anatomy. Disregarding morphological variability may hinder advancements in comprehending valve function and pathology, as well as in enhancing repair outcomes. Therefore, patient-specific computational models represent a valuable tool for advancing both fundamental knowledge and clinical interventions. Stevanella et al. [9] presented a finite-element model of TV using porcine data to design the TV shape model on the basis of their previous studies on the mitral valve. Sensitivity analysis on leaflet mechanical properties were carried out given the lack of experimental data on tricuspid fiber tissue architecture. Kong et al. [10] demonstrated that computational analysis of TR repair by annuloplasty may provide useful insights into patient selections and may serve as pre-operative planning for assessing leaflet coaptation following device implantation. Using ideal TV geometries, Laurence and collaborators [11] developed a highly-detailed computational model of TV that can be easily extended to patient-specific geometries to guide TR repair. Isogeometric-based parametric modeling [12] and fluid–structure interaction (FSI) analyses [13] have also been proposed for the tricuspid valve; however, these approaches primarily focused on the valve apparatus in isolation, without incorporating the right ventricular geometry or enabling a fully patient-specific ventricular–valvular interaction. A detailed review of tricuspid valve biomechanics and modeling approaches can be found in the recent review by Lee and collaborators [14]. Our study builds on the work of Mathur et al. [15], who developed a TV model from an ex-vivo human heart rejected from a transplant and connected to a reperfusion system to keep it alive, enabling mechanical testing and digitization of valve geometry for simulation purposes. However, we extended previous computational models by replicating TV and right ventricular biomechanics in a patient-specific fashion, using computed tomography angiography (CTA) for model personalization. Specifically, we developed an image-based, patient-specific computational framework that integrates tricuspid valve and right ventricular geometry reconstructed from routine CTA, incorporates annular kinematics into finite-element simulations, and combines structural analysis with post-repair hemodynamic assessment following virtual edge-to-edge device implantation. Medical imaging from three patients was utilized to segment the annular and sub-valvular structures, and the resultant finite-element models were created to simulate valve kinematics and biomechanics under physiological and pathological conditions. Simulations of blood flow were carried out to assess the pathological hemodynamic dysfunction associated with TR conditions. The deployment of the MitraClip device was simulated to address the regurgitant valve and assess post-repair RV-related function. This approach advances beyond prior structural-only models by providing a comprehensive evaluation of both valve biomechanics and post-repair flow dynamics. The proposed models offered novel insights into tricuspid valve function and establish a basis for future research on TR in individualized patient scenarios.

## Materials and methods

### Tricuspid valve geometry reconstruction

To reconstruct the TV model, computed-tomography angiography (CTA) images of the patient’s heart were acquired and subsequently segmented by contour lines during both diastole and systole using Mimics medical imaging software (v2023, Materialise, BE). The open configuration of the TV model was regarded as the free-stress configuration and used as the initial condition for biomechanical simulations. Spline curves were utilized to define the saddle-shaped annular configuration for each patient whereas the free edge of the TV leaflets was initially reconstructed by contour lines [15]. All spline curves were subsequently exported as IGES files and imported into the computer-aided design (CAD) software Rhinoceros (v8 McNeel, SA) to reconstruct the TV surface model and sub-valvular structure. The annulus spline was initially unrolled to maintain the physiological extension of the three valve leaflets and the natural annulus curvature. The free leaflet edge contour was modified to conform to the unrolled annulus spline and subsequently refined to mirror the edge profile as demonstrated by ex-vivo [16]. Then, the TV surface was generated by loft protrusion between the unrolled annulus and leaflets free-edge contour. The resulting TV surface was then remapped and rolled onto the segmented TV shape to obtain the final stress-open configuration, using an uniform thickness of 0.78 mm.

**Fig. 1:**
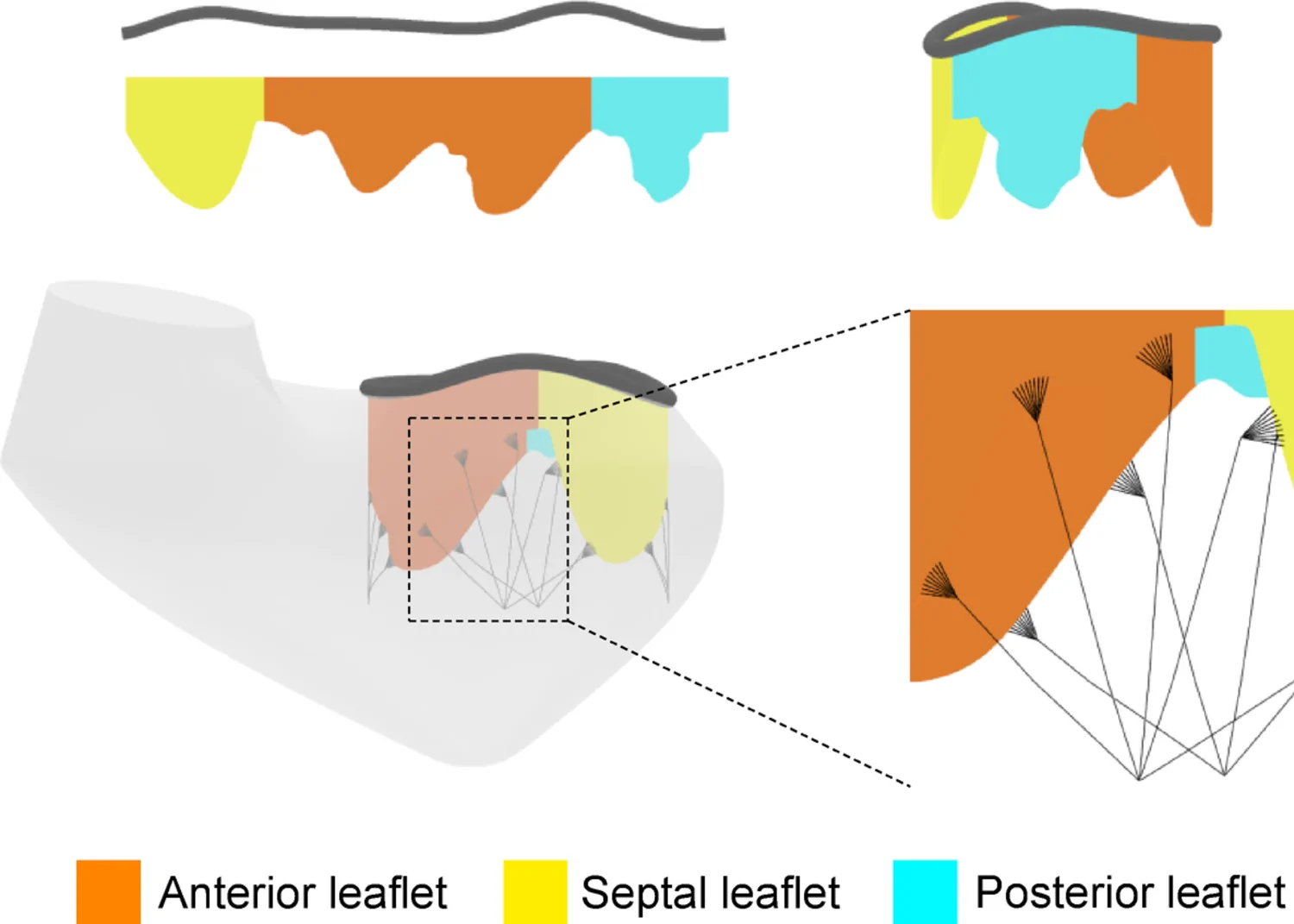
Representation of the unfolded and wrapped geometry of TV as well as the assembly with the RV wall and chordae attachment to each valve leaflet for the patient Case #1

Since chordae tendineae were not clearly visible from CTA images, the ex-vivo data reported by Mathur et al. [15] were used to account for chordae attachment sites to the leaflets and papillary muscles, as well as chordae length and thickness. Several simulations were iteratively performed to refine chordae attachment sites and lengths according to predefined biomechanical criteria, including: (i) continuous leaflet coaptation under physiological systolic loading, (ii) absence of leaflet prolapse or excessive tenting, and (iii) stable stress distribution without element distortion or numerical instability. This approach was kept consistent among all patient-specific models to ensure methodological reproducibility. Figure 1 shows the reconstructed TV model for three patients in both the unrolled and final configurations, emphasizing the saddle shape of the tricuspid valve annulus and sub-valvular apparatus.

Regurgitation was induced by combining geometric and loading alterations commonly associated with functional TR. Specifically, papillary muscle reference points were displaced to simulate subvalvular tethering, and the leaflet free-edge geometry was adjusted to reduce effective coaptation length. In addition, the transvalvular systolic pressure was increased upon 40 mmHg to enhance regurgitant loading conditions. These modifications resulted in incomplete leaflet coaptation and the formation of a residual regurgitant orifice under peak systolic conditions. The virtually-replicated TR condition was repaired using the MitraClip device for each patient model.

### Integration of the right ventricle

A full anatomical representation of RV model was developed by reformatting the CTA images along the RV long-axis. Then, contour lines spaced 1.5 mm apart from the ventricular apex to the distal heart valve plane were generated to depict the endocardial ventricular surface. The splines were imported into CAD software to create a loft protrusion surface between adjacent spline curves. The myocardial wall was not considered, and a shell simplification of the right ventricular wall was utilized instead. While solid wall modeling of ventricular chambers is prevalent in cardiac biomechanics, shell-based approximations have been effectively utilized in simulation studies to diminish mesh complexity and computational expenses while maintaining anatomical accuracy [17].

### FEM analysis of the model

After assembling the TV model with the RV surface, the patient-specific model was exported as IGES file and then imported into ABAQUS/Explicit software (ABAQUS v2023hf2, SIMULIA Inc., Providence, RI, USA). Given the relatively low anisotropy reported for tricuspid leaflets [15], the leaflets were modeled as nearly incompressible, isotropic materials using a two-term strain energy density function with different constitutive material parameters for each leaflet:

1 $$\:W=\frac{{c}_{0}}{2}\left[{I}_{1}-3\right]+\frac{{c}_{1}}{2}[\mathrm{exp}\left({c}_{2}\left[{I}_{1}-3\right]\right)-1]$$

where $\:{c}_{0},\:{c}_{1}$and $\:{c}_{2}$ are material parameter and $\:{I}_{1}$is the strain invariant. Rayleigh alpha damping was employed to account for energy dissipation due to dynamic loading. Table 1 summarizes material descriptors for each leaflets. The chordae tendineae were modeled as truss element with circular cross section of 0.2 mm and using a three-term Ogden material model as shown by Table 2.

**Table 1:** Material parameters of TV leaflets; D = density; α = alpha damping

Leaflet	$\:\boldsymbol{D}\:\left(\boldsymbol{k}\boldsymbol{g}/{\boldsymbol{m}}^{3}\right)$	$\:\boldsymbol{\alpha\:}\left(-\right)$	$\:{\boldsymbol{c}}_{0}\left(\boldsymbol{M}\boldsymbol{P}\boldsymbol{a}\right)$	$\:{\boldsymbol{c}}_{1}\left(\boldsymbol{M}\boldsymbol{P}\boldsymbol{a}\right)$	$\:{\boldsymbol{c}}_{2}\:\left(-\right)$
Anterior	1060	250	1.10	0.12	15.26
Posterior	1060	250	1.10	0.22	31.91
Septal	1060	250	1.10	0.19	2.95

**Table 2:** Ogden material parameters of chordae tendineae

Leaflet	$\:{\boldsymbol{\mu\:}}_{1}\:\left(\boldsymbol{M}\boldsymbol{P}\boldsymbol{a}\right)$	$\:{\boldsymbol{\alpha\:}}_{1}\left(-\right)$	$\:{\boldsymbol{\mu\:}}_{2}\:\left(\boldsymbol{M}\boldsymbol{P}\boldsymbol{a}\right)$	$\:{\boldsymbol{\alpha\:}}_{2}\left(-\right)$	$\:{\boldsymbol{\mu\:}}_{2}\:\left(\boldsymbol{M}\boldsymbol{P}\boldsymbol{a}\right)$	$\:{\boldsymbol{\alpha\:}}_{2}\left(-\right)$
Anterior	0.04	50.40	0.07	16.40	0.15	40.4
Posterior	0.17	49.12	0.12	49.16	0.12	49.16
Septal	0.43	0.11	0.01	0.40	0.02	0.12

Papillary muscle position was accounted in the patient-specific models by two reference points with spatial positions determined by the CTA scan. The reference points were coupled the RV wall by kinematic constraints. As for the attachment sites to the TV leaflet model, the extension of each chordae was iterative optimized to optimal valve closure as defined by continuous leaflet coaptation.

Since this study is not focused on myocardial wall mechanics, the RV wall was modeled as an elastic surface with density of 1060 kg/m^3^. Ventricular contraction was simulated through a fictitious thermal field employing the fluid cavity method in ABAQUS software to observe alterations in ventricular volume. To replicate a physiological expansion, the thermal field was iteratively adjusted to match the diastolic volume measured from CTA imaging for each patient.

After mesh sensitivity analysis, each part of the patient-specific models was meshed using ABAQUS meshing capabilities and element formulation. For the leaflet, hexahedral-dominated solid elements with size of 0.6 mm and reduced-integration were adopted. Shell elements of 2.0 mm were employed for the RV wall whereas truss elements of 0.2 mm were used for chordae. The general contact algorithm enabled the interaction among all parts. The hard contact formulation for the normal direction allowed to prevent element penetration whereas the penalty-based approach with friction coefficient of 0.01 was adopted for tangential behavior among leaflets.

To account for the three-dimensional motion of TV annulus during the cardiac beating, the approach developed previously by our group for the mitral valve was extended [18]. Specifically, the contour lines defining the annulus shape at both systole and diastole were imported in the mathematical language program Matlab (Matlab v2023, MathWorks, CA, USA) and then coordinates of element nodes were projected from the systolic annulus configuration to the diastolic one. This enabled the evaluation of annulus motion, thereby determining the displacement utilized as boundary conditions in the finite-element model to accurately replicate annulus kinematics in a patient-specific manner. Tie constraint was used to couple the distal chordal end to the leaflet nodes. For the TV model, a physiological pressure waveform was applied to the outer leaflet surfaces together with viscous damping to stabilize dynamic oscillations likely occurring during valve closure [19]. All simulations were carried out using mass scaling of 1.0e^− 6^ every 1000 increments to warrant a quasi-static approach.

### The MitraClip device implant simulation

**Fig. 2:**
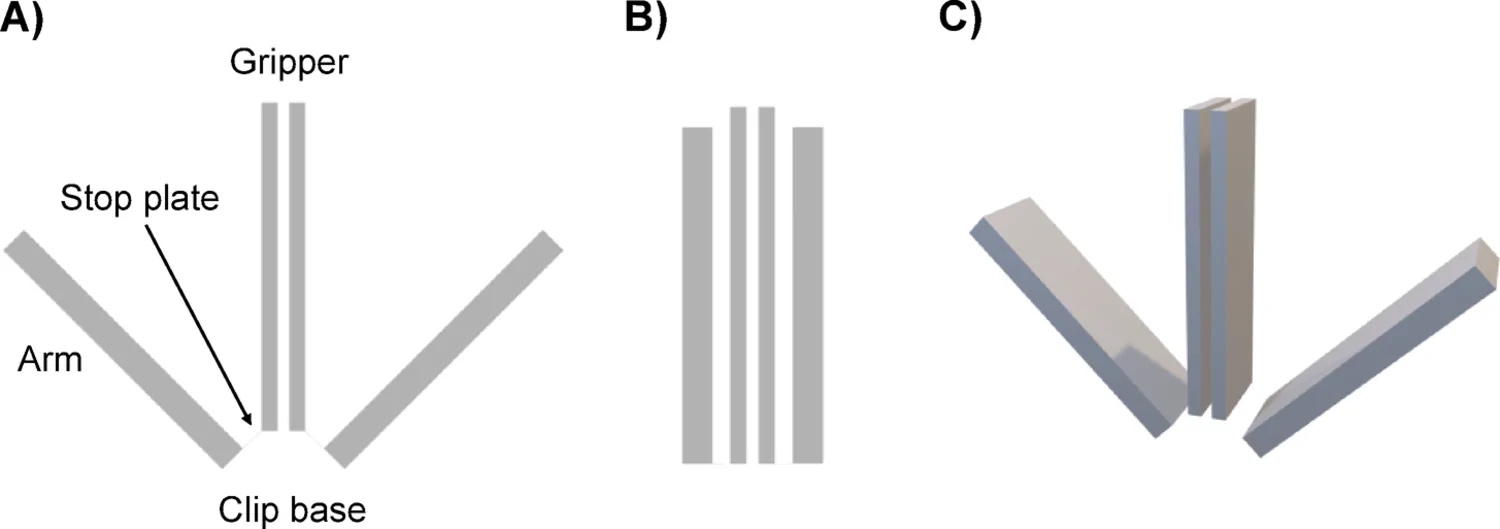
MitraClip device model in the **A** reference and **B** close configurations as well as 3D model representation

As done in a previous study for the mitral valve [19], the MitraClip device was designed in Rhinoceros (v8, McNeel, SA) as a simplified CAD model with four solid plates (see Fig. 2). Specifically, two “arms” with width of 5 mm, length of 16 mm and thickness of 1.5 mm enabled the interaction with the outer leaflet surfaces. The two “grippers” with similar size but thickness of 0.8 mm, interacted with the inner leaflet surfaces. The “stop plate” was modeled to limit the leaflet insertion length as this occurs with the deployment of the MitraClip device.

The CAD model of the MitraClip device was imported in ABAQUS/Explicit as solid rigid part and meshed with element size of 0.3 mm. The arms and grippers can be moved to fully grasp the TV leaflets with respect to a single axis at a hinge point located at the base of the clip. This allowed us defining a general contact between the grippers and inner surface of the leaflet and between the arms and outer surface of the leaflets to replicate the mechanical contact during clip deployment. A reference node was generated not only to align the MitraClip model with respect to the TV, but also for replicating the rotations necessary for capturing the valve leaflets. All degree of freedom were constrained to the reference node. The simulation started with the regurgitant valve and with the arms at the opened configuration. Then, the grippers were rotated by 52 degrees, causing them to face the device arms and thus capturing the TV leaflets. The MitraClip was closed by simultaneous rotation of both grippers and arms with respect to the reference points using a rotation angles as collected from manufacturer guidelines. The final deformed configuration of the patient-specific models was used for computational fluid dynamics.

### Flow analysis

Post-MitraClip hemodynamics were assessed by blood flow analysis using the lattice Boltzmann method in the XFlow software (v2022, Dassault Systèmes, USA). This represents a meshless numerical method particular useful for complex geometries by employing fictive particles that undergo propagation and collision processes over the grid to simulate flow motion under transient and compressible formulation.

Once the deformed configuration of repaired TV model was imported, the internal flow problem was defined by re-orienting the surface normal of each parts towards the interior domain of the ventricular chamber. The blood was modeled as a Newtonian fluid with isothermal properties using temperature of 37 °C, a molecular weight of 30,000 g/mol, dynamic viscosity of 0.0037 Pa x s and density of 1060 kg/m^3^ [20]. Wall-adapting local eddy-viscosity was used as turbulence model. A resolved scale of 0.5 mm was used leading to roughly 750,000 unity D3Q27 lattice particles as determined by sensitivity analysis, whereby the numerically predicted flow velocity across the valve approached the prescribed flow velocity. An automatic time step was utilized to ensure that the numerical speed of sound does not surpass the thermodynamic speed of sound in the flow.

The simulated flow scenario aimed to replicate the flow across the (i) fully-opened healthy TV, (ii) the regurgitant TV and (iii) repaired TV. For the sake of simplicity, only steady state simulations under a zero-pressure flow condition were considered. Specifically, the steady-state simulations were performed to isolate peak flow behavior and enable controlled comparison between configurations, rather than to replicate the full transient cardiac cycle. For the boundary condition, the inflow velocity was set to 0.7 m/s for the healthy and repaired TV models but a three-fold increase was considered for TR condition. No-slip boundary conditions were applied to the TV surfaces and RV wall.

## Results

### FEM simulations

**Fig. 3:**
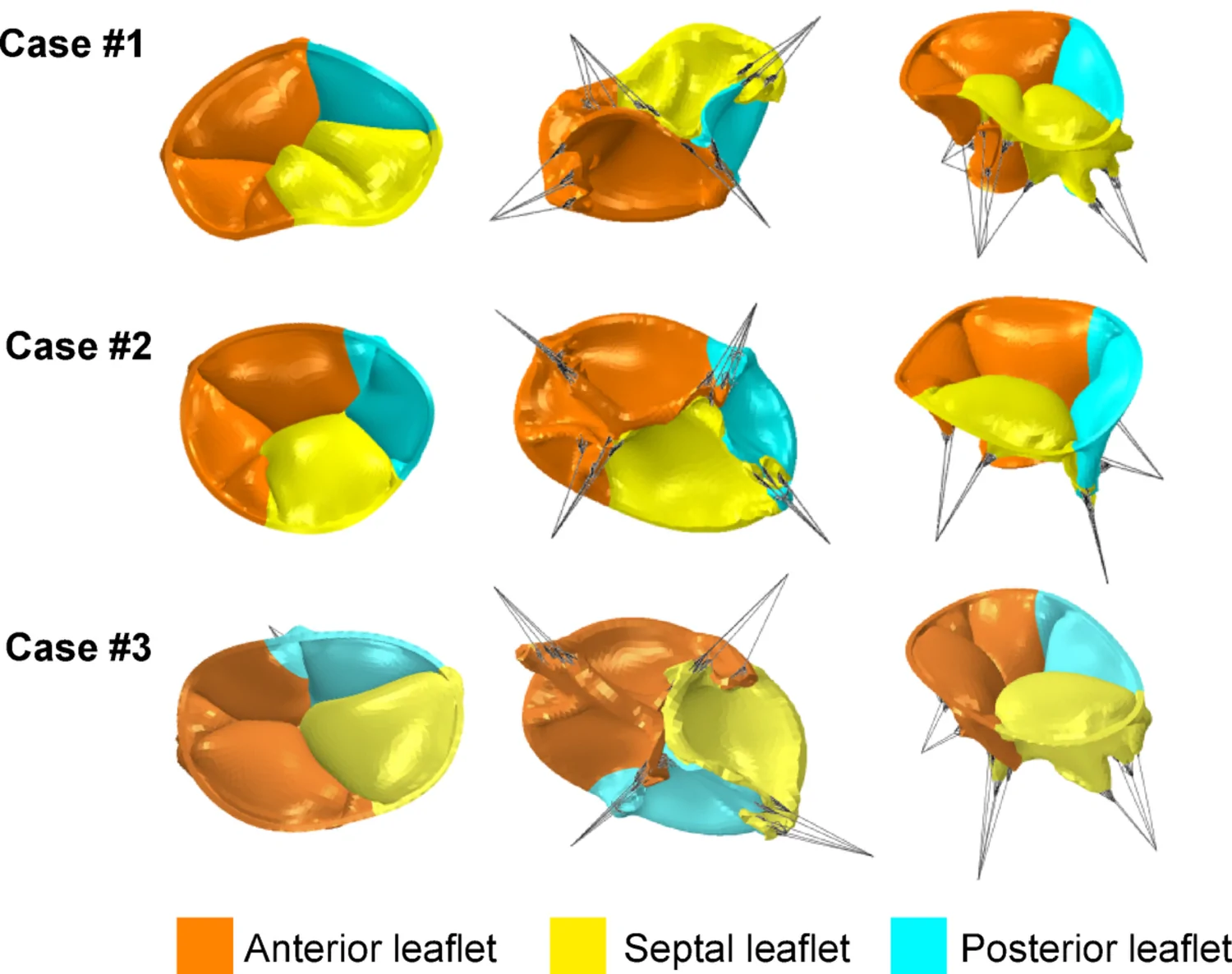
Deformed shape of TV model resulting from each patient simulation at the end-systolic phase of the cardiac cycle

Figure 3 shows the end-systolic closed configuration of the TV for the three patient-specific models following the simulation of the healthy scenario. Valve closure was successfully obtained with the leaflets in contact for sufficient coaptation. This was accomplished through iterative modifications of chordae attachment to the leaflet surfaces and adjustments of chordae length to optimize valve closure. The contraction of the annulus, as modeled by the displacement field from systole to diastole, was essential for valve closure and for producing realistic patient-specific simulations. No significant valve leakage was detected in any of the patient cases.

**Fig. 4:**
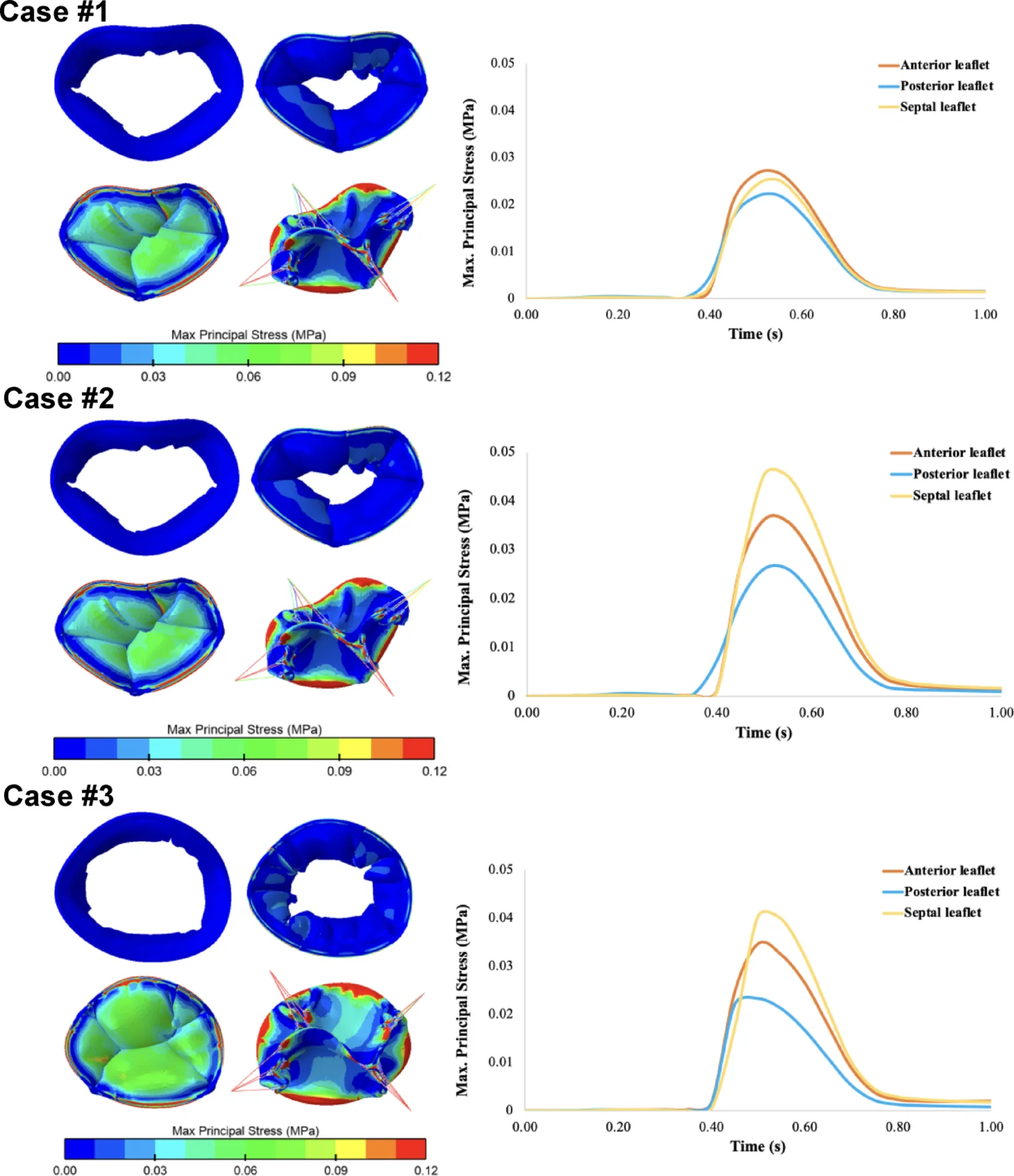
Maximum principal stress distribution at different time step of the cardiac cycle and corresponding stress at mid-height of the leaflet for each patient case

Figure 4 illustrates the distribution of maximum principal stress on the tissue leaflets during three representative phases of the cardiac cycle: the free-stress end-diastolic open configuration, partial closure, and fully end-systolic closed configuration. The profile of maximum principal stress extrapolated at the midpoint of each TV leaflet was acquired throughout the cardiac cycle. Stress values reached their peak at the end-systolic configuration, with small changes between patients. When the tricuspid valve is closed, the chordae tendineae experienced a state of tensile stress. Stress attained its lowest point when the valve was completely opened at the end of diastole.

### Hemodynamic simulation

**Fig. 5:**
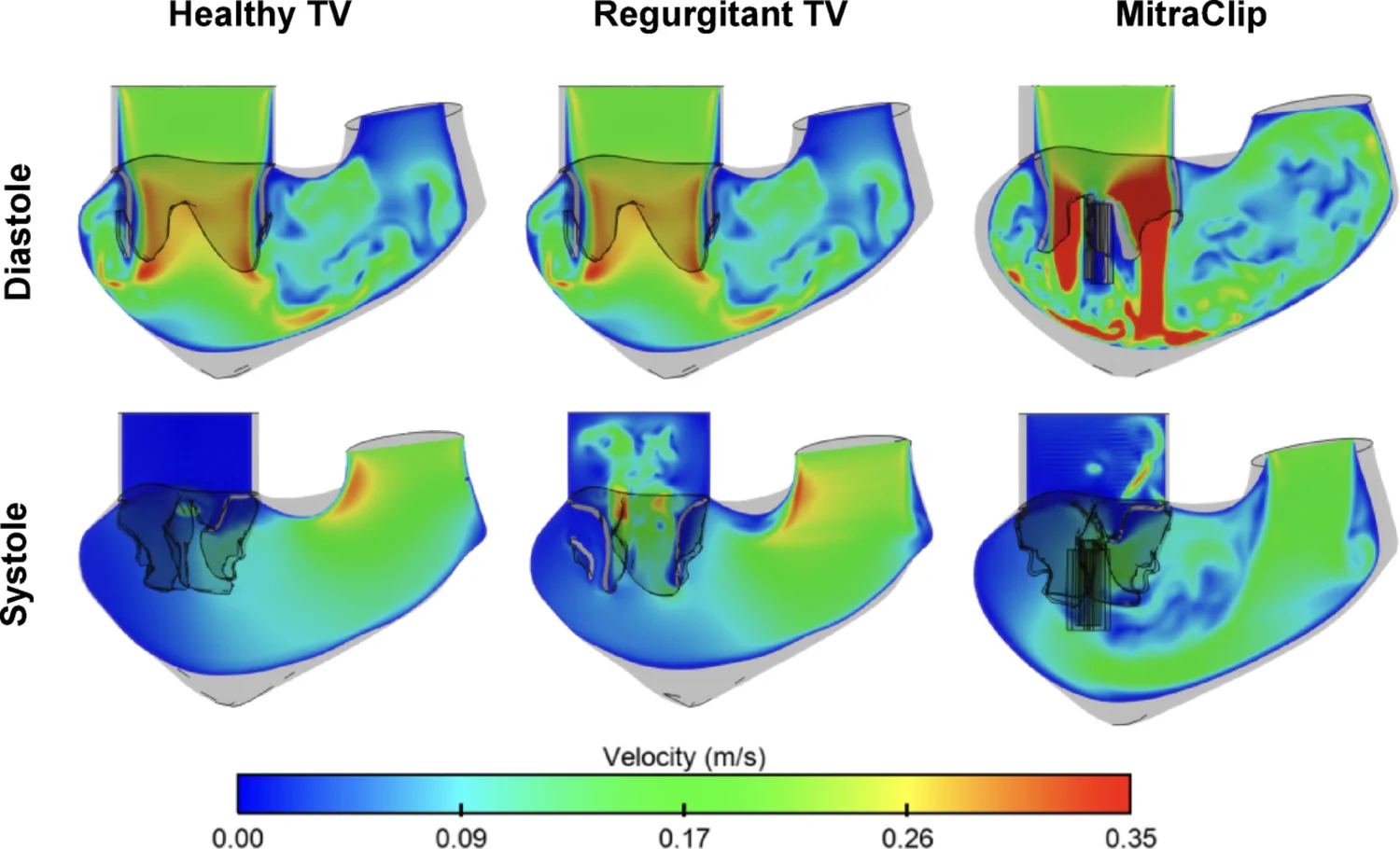
Flow velocity in the RV wall for both the opened (top row) and closed TV models (bottom row) for a representative patient (Case #1) at different hemodynamic scenario

**Fig. 6:**
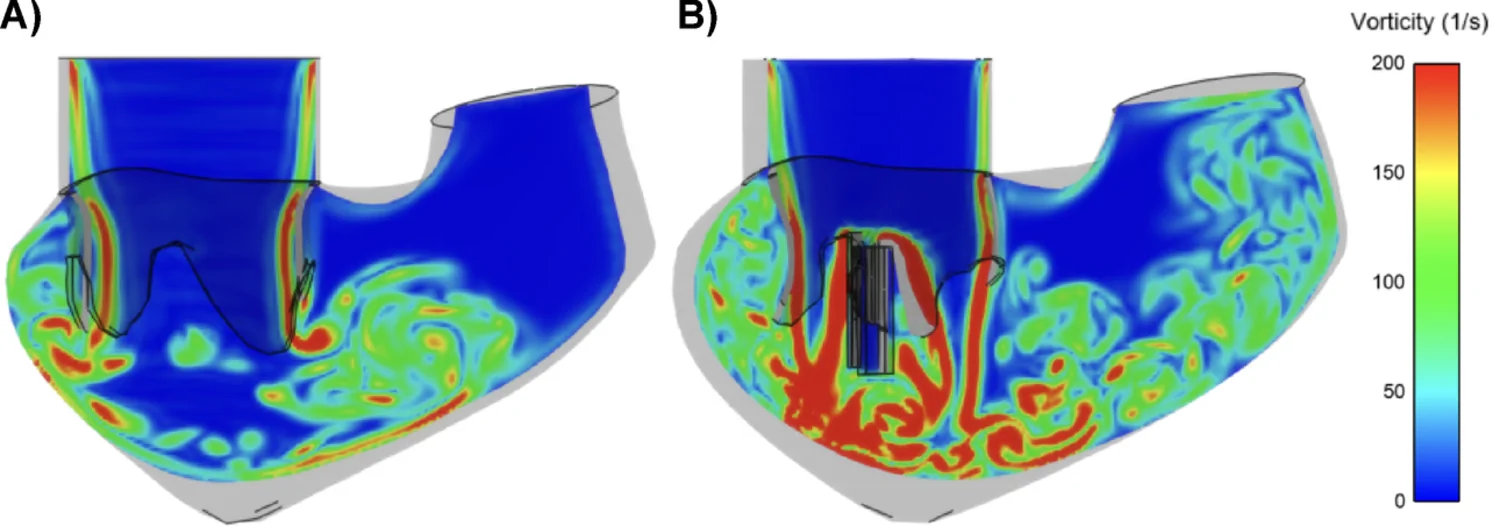
Vorticity map in the RV wall for a representative patient (Case #1) for **A** healthy TV and **B** repaired TV at open valve configuration

Figure 5 depicts the blood flow velocity in the RV wall under both end-systolic and end-diastolic TV configurations for each hemodynamic scenario. The healthy configuration of the TV model demonstrated full closure without backflow across the leaflets. The regurgitant TV exhibited incomplete leaflet coaptation, indicating potential blood leakage from the ventricular chamber to the atrium. Following MitraClip repair of TR condition, the closed valve configuration exhibited no retrograde blood flow. This indicated that the device effectively prevented undesired flow during ventricular systole, likely reflecting a physiologically normal state. Upon activation of the repaired TV, the right ventricular inflow is bifurcated into two distinct flow jets due to the presence of the clip device. The flow split resulted in vorticity near the clip surface, which is notable in comparison to the healthy flow condition. The analysis of the vorticity parameter revealed a localized increase in vorticity magnitude near the clip device, accompanied by a more extensive vortical field throughout the ventricular chamber as shown in Fig. 6. This pattern is likely associated with the reduction in the effective valvular area due to device implantation, portending to flow acceleration and consequently high velocity gradients.

**Fig. 7:**
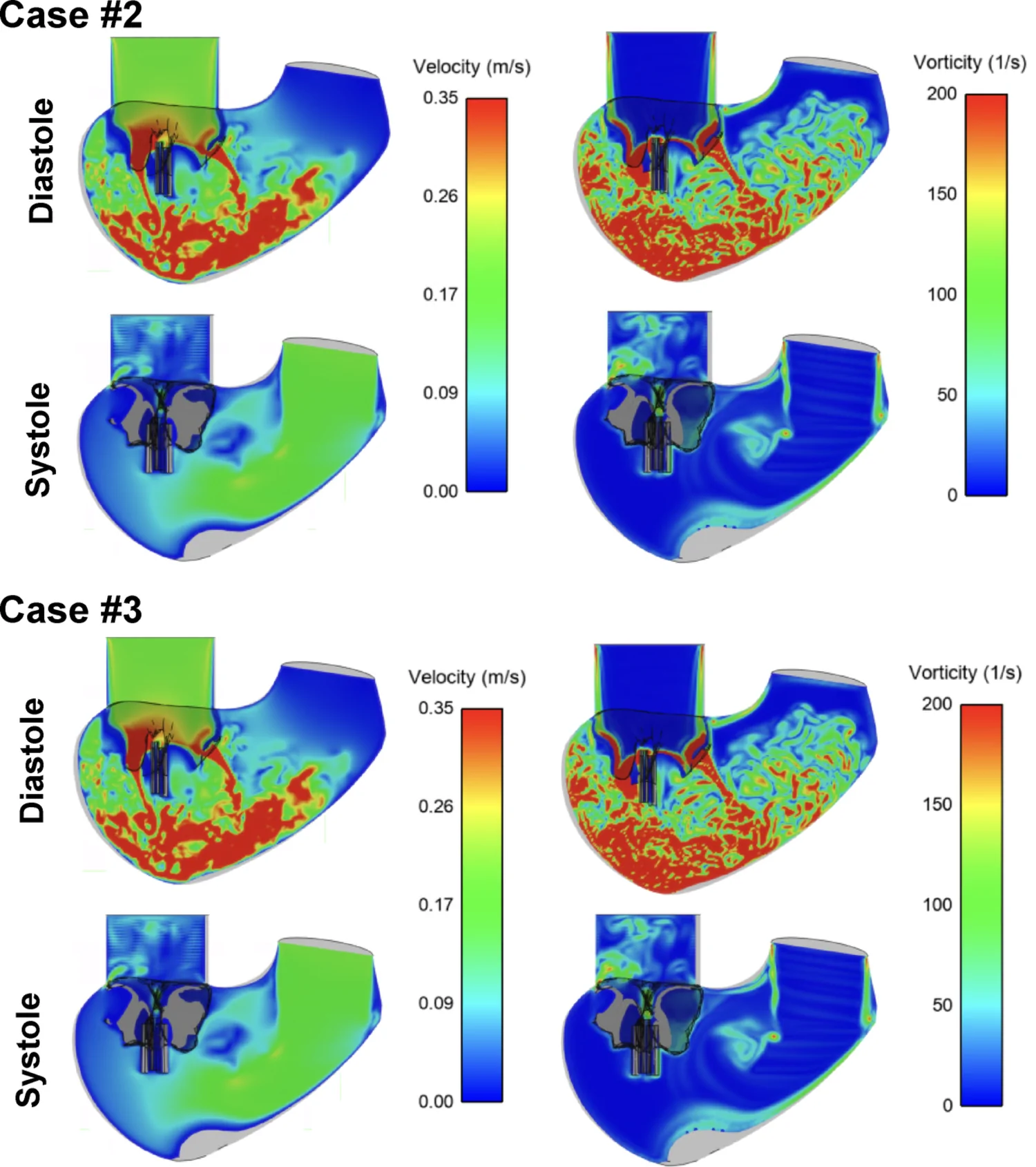
At left flow velocity with valve open (top row) and valve closed (bottom row) of the third model: **a** pathological conditions (regurgitant), **b** after MitraClip implant; at right vorticity inside the right ventricle in healthy conditions (top) and after MitraClip implant (bottom) at open configuration

Comparable post-MitraClip hemodynamic patterns were observed in the other two patient-specific models as shown by Fig. 7. In patient Case #2, a skew flow jet directed towards the pulmonary artery was observed during diastole, with no reverse flow occurring during ventricular systole. Peaks of the vorticity parameter were observed at the apex of the RV wall. Conversely, patient Case #3 exhibited a maximal flow jet aligned with the surface of the clip device on the side adjacent to the pulmonary valve. Vorticity exhibited local maxima along the lateral wall of the right ventricle.

## Discussion

This study aimed to create a patient-specific computational model of the TV using routine medical imaging to model both the valve and RV. The patient-specific TV model was accurately refined to incorporate annulus kinematics during heart beating and to account for realistic leaflet geometries, with the subvalvular chordae apparatus iteratively adjusted to ensure optimal valve closure. The model accurately simulated physiological valve closure and stress distribution throughout the cardiac cycle, effectively representing the complex biomechanical behavior of the TV. Then, the TR condition was simulated and repaired using the MitraClip device to evaluate the hemodynamic status post-implantation. The findings indicated that the captured leaflet reduced tricuspid regurgitant flow during ventricular systole, while creating a vortical field during diastolic filling of the right ventricle wall. This flow patterns may shed light into the long-term performance and durability of the MitraClip device in the setting of TR repair.

We adopted the Grasshopper plug-in to create patient-specific geometries, enabling parametric reconstruction of the TV geometry for flexible adaptation of the annulus and leaflet properties based on individual patient characteristics. Personalized simulations were accomplished by analyzing the valve kinematics throughout the cardiac cycle and by systematically refining the chordae apparatus for optimal valve closure. The simulation of annular kinematics was based by research on the mitral valve and was essential for ensuring complete valve closure, emphasizing the functional interaction between the tricuspid valve and its subvalvular apparatus. Though the calibration of chordae length and attachments was a manual and time-consuming process, this strategy facilitated a realistic closure of TV leaflets by capturing essential individual anatomical details previously unattainable with alternative methods. In comparison to the prior ex-vivo study performed by Mathur and associates [15], which was based on an isolated heart for model reconstruction, the present approach is based on routine clinical imaging for determining the input geometric model. This enables the development of tailored models that can be generated on a patient basis to support the emerging concept of in-silico medicine. The presence of RV wall further extends the physiological realism of the proposed computational framework.

The pronounced increase in the leaflet stress observed across all patient models reflects the physiological loading environment of TV. Stress concentrated in the leaflet belly and near chordal insertion zones and are consistent with experimental and computational data reported in previous studies [10, 15]. This confirms that these regions are the most mechanically subjected tissue area so that the long exposure to such condition may contribute to adverse structural degeneration and regurgitation progression. Using a grow and remodeling principle, Mathur and collaborators [16] suggested that an altered mechanical stress can prevent proper TV coaptation by inhibiting leaflet ability to bend and stretch. They speculated that stress contributes to additional valve dysfunction and that alleviating abnormal loading, enhancing leaflet motion, and decreasing stiffness may restore valve function. Our computational models offer personalized mechanical stress analysis, revealing that stress prediction may indicate mechanical stimuli that activate valve interstitial cells and promote extracellular matrix remodeling. Incorporating patient-specific TV modeling with remodeling principles may provide a means to stratify patients at the risk of maladaptive leaflet al.terations and regurgitation.

Flow simulations complemented the structural analysis by predicting intraventricular flow patterns under both physiological and pathological conditions. For the regurgitant TV configuration, incomplete leaflet coaptation led to retrograde flow consistent with clinical echocardiographic findings of tricuspid regurgitation [1]. After MitraClip deployment, retrograde flow was markedly reduced suggesting restored valve function. These findings corroborates clinical outcomes for clip implantation [7, 8, 21], supporting the ability of patient-specific in-silico models to predict device efficacy. However, flow simulation revealed two divergent flow jets near clip distal surface because of a double orifice configuration as well as an increase in vorticity in the downstream region of right ventricular chambers. Differences were observed from patient-to-patient according to the TV anatomy and device position. To our knowledge, such findings have not previously been reported for the repaired TV, but only observed for mitral valve repair [22]. The presence of two accelerating flow jets may increase local shear gradients. Although the clinical implications of such flow reorganization in tricuspid repair remain to be established, similar phenomena have been associated with altered ventricular remodeling and thrombotic risk in other valvular contexts [22]. The formation of vortices with MitraClip in the downstream area is likely associated with modified pressure distribution in the RV, indicating detrimental remodeling (i.e., dilatation) or pulmonary hypertension. Understanding the impact of MitraClip positioning on RV-related hemodynamics can enhance delivery strategies concerning pressure distribution and ultimately improve patient outcomes. Though this was not here demonstrated, intracardiac vortex dynamics have been recognized as markers of ventricular efficiency and remodeling in cardiovascular disease [23]. Moreover, pronounced local shear gradients in the presence of device-induced flow acceleration have been associated with platelet activation and thrombotic mechanisms in pathological flow environments [22, 24]. Therefore, while our findings should be interpreted cautiously, the observed post-repair flow reorganization may warrant further investigation.

The current study has several limitations. An isotropic formulation was employed for the TV model, despite the anisotropic characteristics of the tissue valve leaflet. The right heart was modeled using a shell material, excluding fiber topology and myocardial active contraction. This resulted from the geometrical challenges in integrating the TV model with the RV model. Furthermore, the absence of a comprehensive fluid-structure interaction analysis limited the capacity to accurately capture transient pressure-flow dynamics and the dynamics of valve closure. This may result in alterations to the predicted flow velocity fields and vorticity patterns. Findings must be confined to the investigated patient anatomies. The limited number of cases reflects the complexity of reconstructing a detailed patient-specific tricuspid valve–right ventricular model from high-quality CTA datasets acquired in both systolic and diastolic phases, which were necessary to account for individualized annular kinematics. Therefore, this study should be considered a proof-of-concept investigation, and future studies including larger and more heterogeneous cohorts are required to extend findings of the proposed framework. Future studies will focus on resolving existing model simplifications by broadening the patient study cohort, enhancing the representation of the RV model, and integrating fully-coupled FSI analyses.

## Conclusion

This study presented a CTA‑based in-silico pipeline that (i) integrates annular motion and tunable chordal architecture into patient‑specific FEM, (ii) simulates a realistic device-based closure for pathological leaflet approximation and (iii) enabled structural and flow assessment in the setting of a regurgitant valve. In all three patients, the healthy simulations demonstrated complete leaflet coaptation and physiologic stress patterns at end‑systolic phase with maxima in the leaflet belly and chordal insertions. Virtual-simulated TR led to incomplete coaptation with retrograde flow, consistent with clinical insufficiency whereas simulated device repair restored competent closure but led to a double‑orifice inflow and localized vorticity. The framework supports pre‑procedural planning by virtually the long-term performance of MitraClip device in patient with TR. Such a patient-specific TR model can improve patient selection, device positioning, and procedural strategy.

## Data Availability

The datasets generated during and/or analyzed during the current study are not publicly available due to ethical issues but are available from the corresponding author on reasonable request.
